# The POETICs of industrial carbon dioxide emissions in Japan: an urban and institutional extension of the IPAT identity

**DOI:** 10.1186/1750-0680-1-11

**Published:** 2006-09-27

**Authors:** Stephan Scholz

**Affiliations:** 1 Global Carbon Project, International Project Office, National Institute for Environmental Studies, 16-2 Onogawa, Tsukuba, 305-8506, Japan; 2 The University of Arizona, Department of Sociology, Social Science Bldg, Room 400 Tucson, AZ 85721, USA

## Abstract

**Background:**

This study applies the POETICs framework (population, organization, environment, technology, institutions and culture) to an analysis of industrial carbon dioxide emissions in Japanese cities. The inclusion of institutional variables in the form of International Council for Local Environmental Initiatives membership, ISO 14001 implementation, and non-profit sector activity addresses the ecological limitations of the often used IPAT (impact = population × affluence × technology) approach.

**Results:**

Results suggest the weak existence of an environmental Kuznets curve, in which the wealthiest cities are reducing their emissions through increased efficiency. Significant institutional impacts are also found to hold in the predicted directions. Specifically, panel and cross-sectional regressions indicate that membership in the International Council for Local Environmental Initiatives and non-profit organizational presence have negative effects on industrial carbon dioxide emissions.

**Conclusion:**

The presence of institutional drivers at the city level provides empirical support for the POETICs rubric, which recasts the ecological framing of the IPAT identity in a more sociological mold. The results also indicate that Japanese civil society has a role to play in carbon mitigation. More refined studies need to take into consideration an expanded set of methods, drivers, and carbon budgets, as applied to a broader range of cases outside of Japan, to more accurately assess how civil society can bridge the issue of scale that separates local level policy concerns from global level climate dynamics.

## Background

The flow of carbon through the Earth's eco-systems is one of the most complex and important of the global cycles. It challenges researchers to be interdisciplinary and synthesize information from both the natural and social sciences in order to understand how it works and what causes it to change. One of the greatest challenges in this task is measuring and accounting for the increasingly skewed contribution of human activities. It is now widely accepted that anthropogenic causes have pushed the atmospheric concentration of carbon dioxide (CO_2_), the main greenhouse gas responsible for global warming and associated climate changes, to its highest level in 420,000 years at 380 parts per million [1].

Rapid population growth coupled with industrialization has clearly been taking its toll. While the developed world has passed through the demographic transition to a low fertility regime defined by an aging population, the developing world is still in the midst of a high fertility regime that is responsible for pushing global population figures upward. In addition to sheer numbers, the concentration of people in urban areas is also increasing rapidly, from 30% of the global population in 1955 to 50% today [32]. In the coming decades, most of the world's population will live in urbanized areas and the greatest urban population growth will take place in developing regions [6].

CO_2 _emissions from energy use, automobile transportation, commodity consumption and cement production in cities are the single greatest perturbation to the carbon cycle [21,22]. This impact could be mitigated if cities chose to follow alternative development paths emphasizing energy efficiency, compact living arrangements and mass transit systems. Either way, cities represent the future of human social organization, which is why the focus of the climate change research community is shifting from burdens of proof at the global level to the generation of actionable knowledge at the urban level.

### Urban and Regional Carbon Management

**Figure 1:**
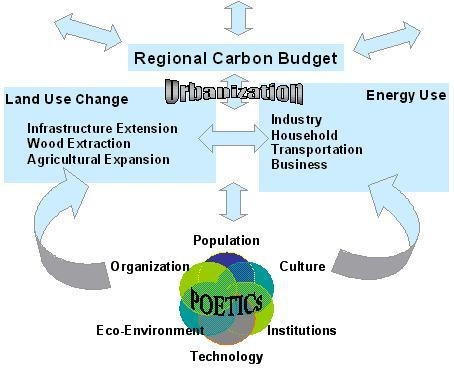
The POETICs of Regional Carbon Budgets [3].

In an effort to better understand the social drivers of CO_2 _emissions the Global Carbon Project of the Earth Systems Science Partnership has established an Urban and Regional Carbon Management (URCM) initiative, which calls for carbon research and management at multiple scales [3]. At the city scale, a carbon budget is theorized to consist of direct carbon emissions from energy use and land-use change, indirect carbon emissions from upstream production embedded in the consumption of commodities, and the sequestration uptake of existing biomass. Figure 1 depicts a regional carbon budget as determined by just the direct emissions of energy use and land-use change. The underlying social drivers are captured by the POETICs rubric, which is a case specific configuration of population, organization, environment, technology, institutions and culture [3].^1^

As presented in Figure 1, most emissions data contained in national greenhouse gas inventories are typically broken down across industrial, residential, transportation and commercial sectors. In this paper I present the results of an attempt to apply the POETICs rubric to an empirical analysis of just industrial emissions at the city level in Japan. This is an extension of previous research on carbon emissions in that it not only tests the classic IPAT identity at a sub-national unit of analysis, but also controls for the effect of more sociologically informed institutional variables.^2^

### IPAT, STIRPAT

Ehrlich and Holdren were the original formulators of the IPAT identity, which has typically been used to assess cross-national variation in environmental impacts [16]. IPAT, as a multiplicative identity, simply states that the impact any particular society will have on its environment is due equal parts to population size and distribution (P), level of affluence (A), and state of technological development (T) [24]. It is sometimes criticized for being an ecological identity that only takes into consideration demographic and economic forces, which stems from its theoretical underpinnings in the heated debate between Neo-Malthusians and Neo-Liberals. To overcome the limitations of this ecological identity as a simple accounting tool with proportional linear effects, Dietz and Rosa first reformulated it as a stochastic model labeled STIRPAT (stochastic impacts by regression on population, affluence, and technology) in 1994 [11]. York, Rosa and Dietz further refined the model and used it to predict carbon dioxide emissions, as well as other atmospheric pollutants, at the cross-national level in 2003 [34]. Through the use of natural logarithm transformed variables York et al. interpret their regression coefficients as elasticities, which are commonly used in econometric models to predict economic outcomes such as price fluctuations. Their ecological elasticities, however, predict the percentage change in a dependent environmental impact variable based on a one percent change in an independent driver variable, when holding all other drivers constant. Other extensions of the IPAT framework that have sampled at the sub-national level include James Cramer's study of air pollution in California and DeHart and Soulé's study of greenhouse gas emissions in North Carolina [7,8]. Both of these studies contend that the IPAT framework can be successfully applied at the sub-national level. In this paper I extend the STIRPAT approach to an analysis of industrial carbon dioxide emissions sampled at the urban level within Japan, and control for the effect of additional institutional variables. These institutional variables are included to address the ecological limitations of the original framework, and take the form of International Council for Local Environmental Initiatives (ICLEI) membership, International Standards Organization 14001 (ISO 14001) certification, and the number of non-profit organizations registered within a city's administrative borders.

### Industrial emissions in Japan

Since World War II, policies in Japan have typically flowed downward from the central government after having been shaped by entrenched and densely connected power interests referred to as the iron triangle of growth (industrial conglomerates, government bureaucrats and the ruling Liberal Democratic Party). Japan was intensely focused on industrial development after 1945, and only through uncontested cooperation within this iron triangle was it able to ascend rapidly to its position as the democratic world's number two economy. This unparalleled achievement came at great cost to human and environmental health, however, which spurred widespread outcry and eventual reform in the 1970s [18]. Notorious disasters, such as Minamata Disease from mercury poisoning in factory runoff, spurred social movements that for the first time in Japan's post-war history were able to exert pressure on the iron triangle [2]. In 1970, the national government had no choice but to pass 14 environmental laws and amendments that moved Japan to the forefront of countries with environmental regulations. Additionally, local governments and industries cooperated to pass pollution control agreements (PCAs) that often exceeded the national government's regulations [18]. These decentralized, bottom-up measures, monitored by local governments, domestic non-profit organizations and an activated populace, successfully held industries accountable for cleaning up manufacturing processes and resulted in widespread gains in technological efficiency.

In the 1990s Japan faced a new set of environmental concerns that were more global in nature. Pressure mounted from the international community to green its aid to developing countries and to help take the lead in mitigating climate change. In hopes of reducing CO_2 _emissions, industries and local governments have therefore been adopting new types of internationally standardized voluntary management plans, such as ICLEI's Cities for Climate Protection Program and ISO 14001. These plans differ from the PCAs of the 70s in that they were developed outside of Japan and do not have the strong mechanisms of locally derived regulatory oversight. Nonetheless, their impact may be more far-reaching because they focus on an industry's entire upstream and downstream lifecycle. The PCAs of the 70s succeeded in implementing technological, end of the pipe solutions, but the driving forces of climate change are so deeply embedded within basic social and economic activities that it calls for a more holistic approach. Whether or not Japan's enthusiastic adoption of these globally diffused and legitimated management plans has met with concrete results still remains to be seen [18]. Through panel and cross-sectional regression analysis I offer an initial and positive answer to this question.

### ISO 14001, ICLEI and civil society

ISO 14001 is a voluntary environmental management standard developed in 1996 by the International Organization for Standardization in Geneva, Switzerland. It addresses all aspects of implementing environmental management systems, including auditing, labeling, performance evaluation and lifecycle assessment. Implementation ensures consistency and comparability in environmental management systems across the globe [26]. As of 2002, Japan had over 8,000 ISO 14001 accredited industries, more than any other country in the world. Local governments have also been seeking accreditation for their office operations to help improve efficiency and to show that they are leaders on environmental issues [31]. When contracting for work, accredited city governments tend to give priority to companies that have also implemented ISO 14001. Certification at the city level therefore has a spillover effect in the submission of bids to environmentally friendly construction, manufacturing, service and waste treatment industries [31].

Shiroi Town, just north-east of Tokyo in Chiba Prefecture, was the first city in Japan to apply for and implement ISO 14001 in 1997. Rapid urbanization and industrialization through the 1980s, and a progressive mayor in the 1990s, influenced Shiroi's government to find new ways to address its severe industrial pollution problems. Reported external benefits of the town implementing ISO 14001 include an improved image of the town and increased acquisition of ISO 14001 certification by other stakeholders and businesses [26]. By 2005, 70 other cities in Japan had been ISO 14001 certified.

ICLEI is an international association of local governments working to improve global environmental conditions by effecting cultural change in city-level organizational operations [17]. Cultural change in this case refers to the re-alignment of core values, assumptions, and symbols of the city government to reflect a desire to operate in an environmentally sustainable manner [28]. The Cities for Climate Change Program is ICLEI's flagship activity, which offers a framework for local governments to reduce greenhouse gas emissions. The framework consists of a five step process requiring participating cities to conduct a greenhouse gas emissions analysis, establish an emissions reduction target, develop a local action plan to reduce emissions, implement the local action plan, and monitor progress and report on results [17]. Cultural change can subsequently occur because these steps break down the barriers to implementation when planners feel that the issue of climate change is too hard to tackle [28]. It also diffuses common environmental values by connecting city planners to an international network of peers who exchange and compare information. Eighteen cities in Japan have become part of the ICLEI network, and much like ISO 14001 certification, they see it as a way to move forward Japan's Environmental Action Plan. The Environmental Action Plan was drawn up in response to the Kyoto Protocol, under which Japan promised to reduce its total carbon emissions to 94% of 1990 levels by the end of the first commitment period in 2012. It stipulates that local governments take responsibility for implementing their own action plans to help honor this commitment.

As a part of this seismic shift in the social and political landscape, new ground is also opening up for civil society. Non-profit organizations (NPOs) have been able to re-establish their foothold and are experiencing a resurgence of activity and legitimacy. It is important to note that the term NPO is used to refer to both domestic and international non-governmental organizations (NGOs). NGO, as the term is typically used in Japan, refers to just international organizations engaged in foreign assistance activities [15].

The watershed event for Japanese NPOs is widely acknowledged to be the Hanshin Earthquake that devastated the Kobe/Osaka region in 1995 [13]. NPOs made great strides in visibility and credibility because they were the first ones on the ground delivering aid and assistance. In contrast, it took days for the top-heavy bureaucracy of the national government to get its act together. Subsequent political fallout led to the passage of the 1998 Law to Promote Specified Nonprofit Activities, which allowed NPOs to incorporate legally for the first time in Japan's history [33]. Domestic social, environmental and economic malaise throughout the Lost Decade of the 1990s, in conjunction with the globalization of standards and the acquisition of legal status for NPOs, has consequently been transforming Japan from "a closed system operating on informal rules where power has been highly centralized, to one that is increasingly transparent, decentralized and allows far greater input from citizens" [20].

Even though it may be premature to find a clear answer, it is rapidly becoming of interest to ascertain whether Japan's rejuvenating civil society is actually meeting with concrete results. Civil society, in the sense that it is used in this paper, resonates with Diamond's notion of the intermediary realm between the private sphere and the state.

*Civil society is the realm of organized social life that is voluntary, self-generating, self-supporting, autonomous from the state, and bound by a legal order or set of shared rules. It is distinct from "society" in general in that it involves citizens acting collectively in a public sphere to express their interests, passions, preferences, and ideas to exchange information, to achieve collective goals, to make demands on the state, to improve the structure and functioning of the state, and to hold state officials accountable*. [10]

An active civil society spurred by the growth of NPOs should be better able to place pressure on local governments to adopt environmental reforms and address citizen concerns, including industrial CO_2 _emissions reform. For example, Shandra et al. in their 2004 study found that countries with greater international non-governmental organization presence also have lower CO_2 _emissions when controlling for the standard PAT variables [24]. I try to reproduce these results at the urban level within Japan, and also investigate the effect of new institutional arrangements such as ISO 14001 adoption and participation in ICLEI's Cities for Climate Protection Program.

### Research question

Direct emissions, as mentioned earlier, are just part of an area's overall carbon budget, which should also take into consideration the indirect carbon emissions embedded in the upstream production of consumer goods, as well as the carbon that is being sequestered in extant biomass. For example, rough estimates of the CO_2 _embedded in Tokyo's purchased consumer goods suggest that its true carbon impact could be as much as three times greater than just the observed, direct emissions [9]. However, because accurate and comparable data on indirect urban CO_2 _emissions don't exist, this paper is limited to an analysis of just direct, industrial emissions sampled in 1990 and 2000. In short, I attempt to answer the following question through panel and cross-sectional regression:

Is there a significant effect of Japanese civil society and new institutional arrangements in the form of NPOs and regulatory initiatives, such as ISO14001 and ICLEI's Cities for Climate Protection Program, on urban level, industrial CO_2 _emissions when controlling for population, affluence and technology?

In answering this question I operationalize the POETICs rubric developed by Penelope Canan for the Global Carbon Project's Urban and Regional Carbon Management Initiative [3]. Canan points out that the full set of drivers accounting for any region's particular carbon profile must be expanded beyond the typical PAT drivers, which essentially constitute a simplistic ecological identity. She therefore recommends also measuring, environmental, institutional and cultural drivers, in addition to the usual population, affluence and technology drivers. In this initial application of the POETICs framework I only focus on the inclusion of additional institutional drivers.

## Results

Table 1 presents the results of the panel analysis, which controls for ICLEI and ISO 14001 effects. Model 1 includes the full set of variables while model 2 is reduced with just the statistically significant variables. The values in parentheses below the actual coefficients represent standard errors.

**Table 1:** City Level Estimates of Industrial Sector Carbon Dioxide Emissions 1990 – 2000: Controlling for ISO 14001 and ICLEI (Fixed Effects Panel)

**Variable**	**Model 1**	**Model 2**
Population	.590 (.538)	
Population Density	-.182 (.474)	
Income	18.539 *** (4.230)	16.947 ** (3.570)
Income Squared	-1.315 *** (.302)	-1.201 *** (.253)
CO_2 _Per Unit Value of Manufactured Shipments	.868 *** (.043)	.869 *** (.043)
ISO 14001	-.011 (.042)	
ICLEI	-.147 * (.080)	-.139 * (.075)
Constant	-58.945 *** (17.619)	-46.643 *** (12.620)

R^2 ^(within)	.807	.805
(overall)	.471	.235
N observations (groups)	256 (128)	256 (128)

* p < .10 ** p < .05 *** p < .01

In the full model both ICLEI and ISO 14001 have coefficients in the hypothesized directions. Only ICLEI membership is significant, however, so I drop ISO 14001 from the reduced model. Population and population density also turn out to be insignificant. This may be an artifact of the fixed effects model as the population distribution within Japanese cities does not change much in the period from 1990 to 2000. In the random effects model, and in the cross-sectional model presented for the year 2000 in Table 2, population effects do come into play when considering variation in the outcome between cases.

**Table 2:** City Level Estimates of Industrial Sector Carbon Dioxide Emissions in 2000 Controlling for NPOs per Capita

**Variable**	**Model 1**	**Model 2**
Population	1.024 *** (.076)	1.016 *** (.077)
Population Density	-.345 *** (.113)	-.253 *** (.087)
Income	1.000 (.723)	
Income Squared	-14.027 *** (3.338)	-13.538 *** (3.450)
CO_2 _Per Unit Value of Manufactured Shipments	.771 *** (.055)	.759 *** (.059)
NPOs Per Capita	-.416 *** (.095)	-.378 *** (.091)
Constant	-2.590 * (1.447)	-2.417 * (1.467)

R^2^	.770	.766
N	191	191

* p < .10 ** p < .05 *** p < .01

**Figure 2:**
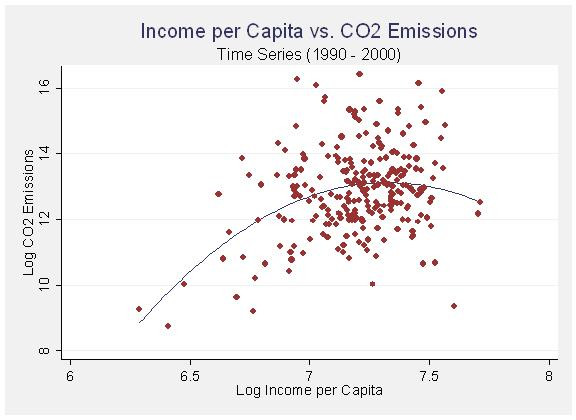
Income per Capita vs. CO_2 _Emissions (Panel 1990 – 2000).

**Figure 3:**
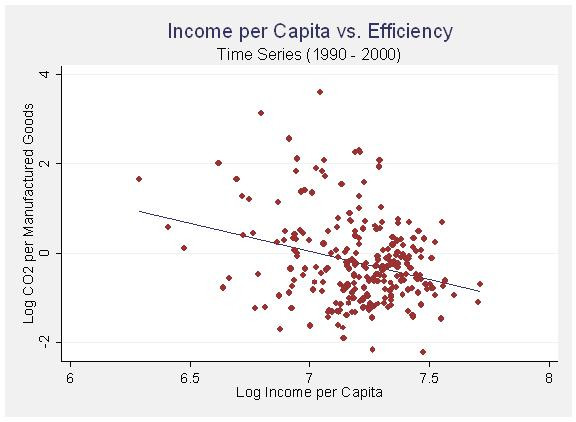
Income per Capita vs. Efficiency (Panel 1990 – 2000).

Income per capita is highly significant and does seem to display an inverted U-shaped relationship. Initial increases in income are associated with dramatic increases in CO_2 _emissions, however, at a certain point this levels off and the effects of income begin to show a modernization effect in which the gains from efficiency actually start to drive emissions back down. The relationship between income and emissions, as well as income and efficiency, are plotted in figures 2 and 3 below with fitted regression lines. Wealthier cities are more efficient and a select few of the wealthiest cities seem to be benefiting from these gains in efficiency by actual reductions in CO_2 _emissions.

It is encouraging to see that an institutional effect in the form of ICLEI membership also remains significant and in the predicted direction when controlling for the other PAT variables. This coefficient cannot be interpreted in the same way as the other log transformed coefficients because it is a dummy variable with the 18 ICLEI cities scored as 1. Nonetheless, from these results, it seems that the reduced model captures a high percentage of the variance in industrial CO_2 _emissions over time (R^2 ^within cases over time = .805) due to just affluence, technological efficiency and ICLEI membership.

Table 2 presents the results of the cross-sectional analysis for cities in Japan in 2000, controlling for non-profit organizations per capita. Once again, both full and reduced models with robust standard errors in parentheses are presented, and unlike the panel analysis, all of the variables remain significant.

**Figure 4:**
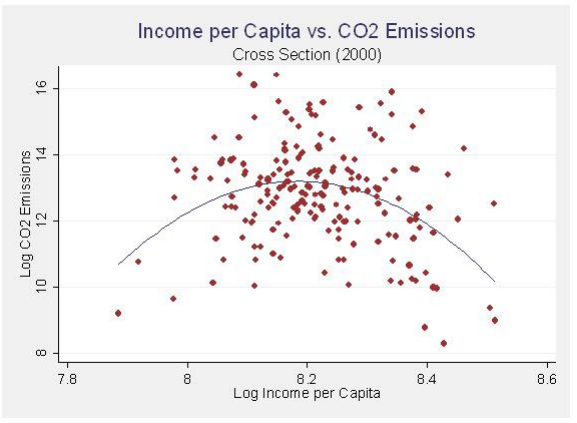
Income per Capita vs. CO_2 _Emissions (Cross Section 2000).

**Figure 5:**
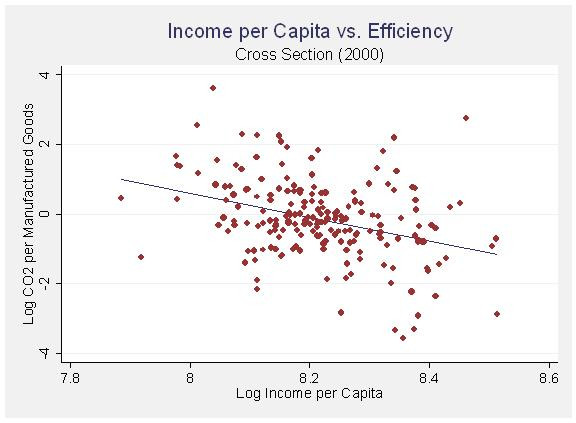
Income per Capita vs. Efficiency (Cross Section 2000).

In this model, which takes into consideration variation between cases instead of variation within cases over time, population and population density both have effects in the predicted directions. City size in terms of population correlates with higher emissions while population density correlates with lower emissions. Although the linear income per capita variable is insignificant, the quadratic transformation is significant and in the predicted negative direction. Once again I display the relationship graphically with fitted regression lines in figures 4 and 5. When looking at the variation between cities as opposed to variation within cities over time, the effect of income displays a much stronger ecological modernization effect. The wealthiest cities clearly seem to be reaping the benefits of increased efficiency.

It is again encouraging to see that a civil society/institutional effect remains in the reduced equation, with a one percentage increase in NPOs per capita corresponding with a .373% decrease in emissions. The reduced model accounts for a high percentage of the variance in the outcome with an R^2 ^of .766.

## Discussion and conclusion

In this paper I present an initial analysis of progressive institutional arrangements on industrial CO_2 _emissions in Japanese cities. Although it is a coarse grained extension of the STIRPAT approach to urban level environmental impacts, statistically significant effects of ICLEI membership and NPOs per capita are found to hold when also controlling for population, affluence and technology. These results therefore lend credence to the POETICs rubric developed under the Global Carbon Project's Urban and Regional Carbon Management Initiative, which calls for carbon research and management at multiple scales through the use of an extended and more sociologically informed set of drivers [3]. The results also support the contention that Japan's burgeoning civil society is beginning to have real impacts on organizational behavior and environmental outcomes.

However, in developed countries such as Japan, where there has been some success in cleaning up industry and making energy production more efficient, the gains may be offset by increasing household consumption and travel. Japanese cities have seemingly gone through the type of transition theorized by ecological modernization. The industrial sector in wealthier cities has made a successful and concerted effort to clean up its act, but this is deceptive because dirty manufacturing can simply relocate to other areas of the world. This, in combination with increased consumption, means that cities in developed countries like Japan still face severe environmental challenges [23].

Future studies need to expand beyond Japanese cities to include, especially, the cities and mega-cities of the developing world. These cities will account for the bulk of global population growth and may never attain the level of wealth found in Japanese cities. It is therefore unreasonable to expect that economic development will have the same kind of modernization effect in terms of actually reducing the scale of emissions.

Other methods also need to be employed that are able to systematically compare a richer set of drivers accounting for the variation in CO_2 _emissions across multiple sectors, and not just the industrial sector. For example, it would be of interest to see if new institutional arrangements, such as ICLEI membership, ISO 14001 certification and NGO activity, configure differently with varying outcomes depending on a city's level of corruption or dependence on foreign direct investment. Experience suggests that carbon emission mitigation is most likely to be supported when it is linked to high priority local issues and when local governments have the power to shape their own policies [21,22]. Full carbon budgets for cities also need to be calculated that go beyond simple emission profiles to include embedded carbon in the consumption of products and the sequestration potential of surrounding biomass. Such a refined and holistic approach would enable more sophisticated planning at the urban level to meet the ever-growing need for global carbon mitigation.

## Methods

### Cases included

The initial population in this study was intended to be Japan's 200 largest cities. During the course of the analysis, however, cities with missing information were discarded yielding a final N of 128 for the panel analysis and a final N of 191 for the cross-sectional analysis. In the panel analysis I test for the presence of ISO 14001 and ICLEI effects across two points in time, 1990 and 2000. A dummy variable is used to score those cities that had been ISO 14001 certified or joined the ICLEI network. I run a separate, ordinary-least squares regression for the year 2000 testing for an effect of NPO presence. Only one outlier, the city of Kurashiki, which is more than three standard deviations above the mean on the dependent variable of industrial CO_2 _emissions, was eliminated.

### Dependent variable

#### Industrial CO_2 _emissions per City

As the dependent variable in this study I use the amount of industrial CO_2 _emissions produced per city. The emissions data for the year 2000 are estimates taken from an environmental assessment report compiled by The Coalition of Local Governments for Environmental Initiative [5]. In 2005, COLGEI released a white book report on industrial emissions for each city in Japan broken down across 35 classifications such as food and beverages, textiles, plastic products, rubber products, iron and steel, machinery, fabricated metal products and so forth. The number and size of industrial establishments per city is compiled and CO_2 _emission coefficients are applied to calculate emission estimates based on the type and amount of fuel used for production, heating and cogeneration, and electricity consumption [5]. Independent researchers, Shibata and Nakaguchi, using the same methodology, calculated the emission estimates for 1990 and graciously made them available for this analysis [25]

### Independent variables

#### Population

Two measures of population are controlled for in this study: the size of a city in terms of sheer numbers, and density. City size should correlate with greater CO_2 _emissions, whereas population density, representing more compact building arrangements, should be negatively correlated.

#### Organization (affluence)

Organization, in the sense intended by the URCM framework, refers to a measure of stratification within a region's social, economic or political structure [3]. In this study I use the more traditional formulation from the IPAT identity by employing average income per capita. Cross-national studies of CO_2 _emissions have typically controlled for a measure of affluence to see if there is an ecological modernization effect. When plotted, this relationship should take the shape of an inverted U, the so-called environmental Kuznets curve, as emissions are theorized to increase with initial economic development and decrease once a certain level of modernization is reached. When comparing cities within Japan a similar type of relationship may exist, although it could be possible that they are all situated on the backside of the curve due to their uniformly high levels of development. The most affluent cities should still exhibit lower industrial CO_2 _emissions, however, because they have less polluting, post-industrial, information technology modes of production. Wealthier cities may also have a more educated and environmentally conscientious citizenry. I use average income per capita to see if there is a linear relationship between affluence and emissions, and average income per capita squared to test for an ecological modernization effect.

#### Technology

In York et al.'s cross-national application of the STIRPAT model, they find technological efficiency to be a significant driver accounting for the variation in emissions. However, they also find that efficiency does not operate independently of scale. Therefore, the wealthiest nations, which are the most efficient, do not benefit enough from these gains in efficiency because they are still by far the greatest CO_2 _emitters in terms of overall scale [34]. In this study I employ an efficiency measure by dividing industrial CO_2 _emissions per city by the amount of revenue generated from manufacturing shipments in that city's industrial sector.

#### Institutions

Institutional effects are measured via a city's participation in ICLEI's Cities for Climate Protection Program and ISO 14001 certification. This is scored as a dummy variable with 70 cities having been ISO 14001 certified and 18 cities having joined with the ICLEI network. I also use the number of NPOs in a city per capita as a proxy for civil society engagement. Presumably, a greater amount of NPOs per capita should correlate with lower industrial CO_2 _emissions. Taken together, all three of these variables constitute the institutional environment for the purpose of this analysis.

#### Regression diagnostics

For the panel analysis I am only able to include data taken on two points in time, 1990 and 2000, and test for the effects of implementing ICLEI or ISO 14001 environmental management plans. Both fixed effects and random effects models estimate coefficients of similar magnitude and direction. The random effects model, taking into consideration both between case variation and within case temporal variation, should be more efficient and does in fact have a higher R^2^. I only present the estimations from the fixed effects model, however, because the asymptotic assumptions of the Hausman test failed to be met [14]. The fixed effects model applies ordinary least squares regression to only the differences within each city across time, but not between cities at the same points in time, to calculate estimates. Although it is possible that a random effects model might still be appropriate, I can be confident that the fixed effects model will, in any case, be more consistent. Cases that have missing or insufficient data on any of the dependent or independent variables were eliminated.

For the ordinary least squares regression analysis I test for the effect of non-profit organizations per capita in just the year 2000. I could not include this civil society variable in the panel analysis because there is no official information on non-profit organizations prior to 1998. Multicollinearity in the cross-sectional analysis does not seem to be a problem as the variance inflation factor scores never exceeded a value of ten. In both the panel and cross-sectional analysis I eliminate the city of Kurashiki as an extreme outlier that is three standard deviations above the mean on CO_2 _emissions. All coefficients in both analyses, except for the dummy variables, are also presented as natural logarithm transformations, and standard errors are robust.

## Appendix

1. For other reformulations of the IPAT identity see Shandra et al'*s *extension to include NGOs at the cross-national level [24], and Catton and Dunlap's original re-working of the classic POET framework [4] developed by Duncan [12].

2. For other research that analyzes city level carbon dynamics and their impact on the global carbon cycle please refer to Svirejeva-Hopkins and Schellnhuber's recent ecological modeling approach [29,30].

## Competing interests

The author(s) declare that they have no competing interests.

## Abbreviations

CO_2 _– Carbon Dioxide

COLGEI – Coalition of Local Governments for Environmental Initiative

ICLEI – International Council for Local Environmental Initiatives

IPAT – Impact = Population × Affluence × Technology

ISO 14001 – International Standards Organization 14001

NPO – Non-Profit Organization

NGO – Non-Governmental Organization

PCA – Pollution Control Agreement

POETICs – Population, Organization, Environment, Technology, Institutions and Culture

STIRPAT – Stochastic Impacts by Regression on Population, Affluence and Technology

URCM – Urban and Regional Carbon Management
